# Cell Type-Specific Transcriptomics of Lateral Root Formation and Plasticity

**DOI:** 10.3389/fpls.2019.00021

**Published:** 2019-02-12

**Authors:** Annika Kortz, Frank Hochholdinger, Peng Yu

**Affiliations:** INRES, Institute of Crop Science and Resource Conservation, Crop Functional Genomics, University of Bonn, Bonn, Germany

**Keywords:** fluorescence activated cell sorting (FACS), laser capture microdissection (LCM), lateral root, pericycle, transcriptome

## Abstract

Lateral roots are a major determinant of root architecture and are instrumental for the efficient uptake of water and nutrients. Lateral roots consist of multiple cell types each expressing a unique transcriptome at a given developmental stage. Therefore, transcriptome analyses of complete lateral roots provide only average gene expression levels integrated over all cell types. Such analyses have the risk to mask genes, pathways and networks specifically expressed in a particular cell type during lateral root formation. Cell type-specific transcriptomics paves the way for a holistic understanding of the programming and re-programming of cells such as pericycle cells, involved in lateral root initiation. Recent discoveries have advanced the molecular understanding of the intrinsic genetic control of lateral root initiation and elongation. Moreover, the impact of nitrate availability on the transcriptional regulation of lateral root formation in Arabidopsis and cereals has been studied. In this review, we will focus on the systemic dissection of lateral root formation and its interaction with environmental nitrate through cell type-specific transcriptome analyses. These novel discoveries provide a better mechanistic understanding of postembryonic lateral root development in plants.

## Introduction

A key strategy of plants to adapt to changing moisture and nutrient availability in soil is the formation of a postembryonic root system. Among postembryonic root types, lateral roots are all roots that emerge from other roots. Highly branched lateral roots absorb most of the soil resources via their large root surface (Yu et al., 2016b). The genetic control of lateral root development in the dicot model plant Arabidopsis (Lavenus et al., 2013) and in monocot cereals (Yu et al., 2016b) has recently been reviewed. RNA sequencing (RNA-seq) has been widely adopted to study the transcriptomic landscape of organ and tissue development in plants (Stelpflug et al., 2016). Transcriptome analysis of lateral roots has been shown to be distinct from that of parent roots in both maize and rice, supporting the notion that fine lateral roots and thicker roots are functionally different (Gutjahr et al., 2015; Yu et al., 2018). Comparative transcriptome analyses of wild-type and mutant root systems in maize and rice have revealed molecular functions involved in auxin signaling and cell cycle regulation during lateral root formation (Zhang et al., 2014; Zhao et al., 2015). However, organs or tissues are composite structures consisting of multiple cell types with different biological functions and thus disparate transcriptome profiles (Schnable et al., 2004).

Transcriptomic analyses of single tissues, single cell types and in its extreme, single cells, have been successfully introduced in medical research and have subsequently been adopted in plant science (reviewed in: Gautam and Sarkar, 2015). There are two main systems that have been utilized in sampling single cell types and single cells. First, fluorescence activated cell sorting (FACS) was initially introduced as a tool to analyze mammalian blood cells (Hulett et al., 1969). This technique requires that cells flow in a fluid stream through the focus of a light source, where detectors can distinguish between different properties of these cells (Galbraith, 2010). Subsequently cells are separated either based on their endogenous or artificially added fluorochromes, or by metric properties estimated from their absorption and scattering behavior (Galbraith, 2010). By using multiple cell type-specific reporters such as green or yellow fluorescent proteins, distinct cell types of a single tissue can be sampled separately (Iyer-Pascuzzi and Benfey, 2010). A drawback of FACS is the requirement of cell type-specific marker lines, which are only available for selected model organisms. Moreover, these marker lines must be protoplasted by enzymatic digestion of the surrounding cell walls, to allow subsequent separation of different types of cells by the FACS system (Galbraith, 2010).

The second technique used for cell type-specific and single cell isolation is laser capture microdissection (LCM). This approach was initially developed to isolate specific tumor cells from infested tissue without compromising their DNA, RNA, and protein integrity (Emmert-Buck et al., 1996). During LCM, the cells of interest are visualized and selected through a light microscope and subsequently excised with a pulsed infrared laser (Nelson et al., 2006). This allows the isolation of specific cells from complex tissue samples comprising distinct cell types (Gautam and Sarkar, 2015). In contrast to FACS, LCM is not confined to cell suspension. Nevertheless, an elaborate sample preparation is still needed to ensure the high quality and integrity of the cells under analysis (Ludwig and Hochholdinger, 2014). The application of these two cell sorting techniques, in combination with downstream transcriptome analyses in lateral root research, will be summarized in the following chapters of this review.

## Cell Type-Specific Transcriptomics Targeting Lateral Root Formation in Arabidopsis

In Arabidopsis, only cells of the pericycle cell layer divide and contribute to lateral root formation (van Norman et al., 2013). Therefore, the pericycle is the most promising target of cell-specific transcriptomics to study lateral root development in this species. The first cell type-specific transcriptome atlas of the Arabidopsis primary root was created by a combination of FACS and microarray experiments (Birnbaum et al., 2003). In this study, stele, endodermis, endodermis plus cortex, epidermis, and lateral root cap cells from the meristematic zone, elongation zone, and differentiation zone were separated and the transcriptomes of these cell and tissue types were analyzed (Birnbaum et al., 2003). This approach did not directly aim at deciphering lateral root development because the pericycle, as the origin of lateral roots, was not separately collected but included in the stele tissue sample. However, it paved the way for cell type-specific transcriptomic analyses and served as a valuable data source for further analyses. To further refine the resolution of cell type-specific expression in roots, the transcriptomes of 14 root cell types, including lateral root primordia, whole pericycle, phloem pole pericycle cells, and xylem pole pericycle cells, were analyzed separately for thirteen regions along the longitudinal root axis in a follow up study. This allowed detecting expression variation between different developmental stages by the combination of manual dissection, FACS and transcriptome profiling via microarray (Brady et al., 2007).

In Arabidopsis, lateral roots initiate from pericycle cells located at the xylem poles (Dolan et al., 1993; Parizot et al., 2008). The usage of two distinct marker lines for xylem and phloem pole cells in FACS experiments enabled the search for variations of the transcriptomic landscape within these distinct pericycle cell types. With this setup, the auxin biosynthetic genes *CYP79B2, CYP79B3, SUPERROOT1*, and *SUPERROOT2* were found to be enriched in lateral root primordia and phloem pole pericycle cells, while tryptophan-dependent auxin biosynthetic genes were enriched in the lateral root primordia and in pericycle cells (Brady et al., 2007). Moreover, FACS sorting of xylem pole pericycle cells of Arabidopsis and subsequent transcriptome analyses, resulted in 1,920 genes differentially expressed at different stages of mitosis (De Smet et al., 2008). Fifteen of these genes were identified as putative key regulators of asymmetric cell division and as being involved in the specification of cell fate during the initiation of lateral roots. Among those, *ARABIDOPSIS CRINKLY4* (*ACR4*, AT3G59420), which encodes a membrane-localized receptor-like kinase, is specifically expressed in the daughter cells, which arose from the first and second asymmetric division of the pericycle cells. This suggests a role of *ACR4* in the regulation of pericycle cell division during the initiation of lateral roots and inhibition of division of neighboring cells (De Smet et al., 2008).

In an effort to utilize transcriptomic data from multiple root cell types generated in different experiments, a spreadsheet application termed VisuaLRTC has been introduced (Parizot et al., 2010). Using this tool, the authors explored the expression of members of the Aux/IAA, AUXIN RESPONSE FACTOR (ARF) and LATERAL ORGAN BOUNDARIES DOMAIN (LBD) families, which are involved in auxin signal transduction. The authors applied multiple criteria including for instance SLR-1 and ARF7-ARF19 dependency of expression, or xylem or phloem pole pericycle expression (Parizot et al., 2010). Through this approach, the authors detected known regulators of auxin signal transduction such as *IAA29, IAA5, IAA11*, *IAA2, LBD17*, and *LBD33* as promising candidates for lateral root development (Parizot et al., 2010). Moreover, they discovered 211 additional candidate genes for lateral root development including known regulators of lateral root development (Parizot et al., 2010). Furthermore, they identified 19 pericycle-specific genes. Most of them were of unknown function and nearly all were down regulated by auxin (Parizot et al., 2010). In another *in silico* transcriptome analysis of pericycle cell populations and their associated vascular phloem or xylem poles tissues, only minorities of genes were either enriched in phloem pole or xylem pole pericycle cells (Parizot et al., 2012). Furthermore, these researchers observed a substantial overlap between the expression of pericycle cells and their respective adjacent vasculature tissue. In phloem pole pericycle and phloem pole cells, this overlap was higher than in xylem pole pericycle cells and xylem pole cells. The authors explained this observation by the meristematic identity of the xylem pole pericycle cells, which distinguishes their function from the adjacent phloem pole pericycle cells (Parizot et al., 2012).

## Cell Type-Specific Transcriptomics Targeting Lateral Root Formation in Monocots

In contrast to Arabidopsis, lateral root primordia of maize and rice are formed from two different cell types: pericycle and endodermis cells (Bell and McCully, 1970; Clowes, 1978). Furthermore, in maize, lateral roots emerge from phloem pole pericycle cells compared to xylem pole pericycle cells in Arabidopsis [reviewed in: Hochholdinger and Zimmermann (2008)].

In rice (*Oryza sativa*), the combination of LCM and microarray profiling was used to create a cell type-specific transcriptome atlas of 40 cell types from shoot, root and germinating seeds at several developmental stages. The aim of this study was to identify cell type-specific genes and gene classes as well as cell-specific promoter motifs (Jiao et al., 2009). Through the application of LCM-assisted microarray experiments the transcriptome of three cell type clusters: (1) epidermis, exodermis and sclerenchyma, (2) cortex, and (3) endodermis, pericycle, and stele termed endstele were compared in rice crown roots, at different developmental stages. In total, 232 genes were up regulated in cluster (3) comprising endodermis, pericycle and stele cells in comparison to the other two clusters and in comparison to stele tissue collected further away from the root tip (Takehisa et al., 2012). 71 genes associated with cell division and elongation as well as numerous transcription factors were only expressed in the region of 1–3 mm from the root tip emphasizing a role particularly in priming and initiation of lateral roots (Takehisa et al., 2012). An *AP2/EREBP* gene (Os07g0669500), which was assorted to this group, is a homolog of Arabidopsis gene *PUCHI*. The *PUCHI* gene is involved in specific patterning of cell division during the early stages of lateral root primordium development (Hirota et al., 2007). Another member of this gene cluster (Os03g0659700) shares similarities with *Arabidopsis AS2-LIKE 18* (*ASL18/LBD 16*), *ASL16/LBD29*, *ASL20/LBD18*, and rice *CROWN ROOTLESS 1/ADVENTITIOUS ROOTLESS 1* (*CRL1/ARL1*), which are linked to lateral root initiation (Inukai et al., 2005; Okushima et al., 2007; Lee et al., 2009; Coudert et al., 2015). In addition, the homolog (Os09g0531600) of the Arabidopsis gene *LATERAL ROOT PRIMORDIUM 1*, which has been previously linked to the early development of the lateral root meristem (Smith and Fedoroff, 1995), was specifically expressed in the endodermis, pericycle and stele cluster (Takehisa et al., 2012). Furthermore, the preferential expression of auxin biosynthesis and signaling-related genes in the endstele tissue that is positioned 1–3 mm away from the root tip was observed in this study, supporting the notion of an auxin-associated mechanisms of lateral root initiation similar to Arabidopsis (Takehisa et al., 2012).

In maize (*Zea mays*), LCM was utilized to isolate pericycle cells from primary roots of the lateral root deficient mutant *rootless with undetectable meristem 1* (*rum1*) and subjecting them to a subsequent transcriptome comparison with wild type pericycle cells. In this study, 90 genes preferentially expressed in wild type and 73 genes preferentially expressed in *rum1* pericycle cells were identified (Woll et al., 2005). Among the annotated genes, genes involved in signal transduction, transcription, and cell cycle were identified, indicating a link between these protein coding genes and lateral root initiation (Woll et al., 2005). Two of the genes preferentially expressed in wild type pericycle cells were helix-loop-helix transcription factors (NP_194827.1 and AAO72577.1), known to control the proliferation and development of specific cell lineages (Heim et al., 2003) undermining their possible involvement in lateral root initiation. In this study, the gene *Cyclin T2* (BAD17160.1), which is involved in the regulation of eukaryotic cell cycle progression was up regulated in wild type pericycle cells (Wang et al., 2004).

To survey the transcriptome of pericycle cells of the maize primary root before lateral root initiation, pericycle and non-pericycle cells isolated via LCM were compared (Dembinsky et al., 2007). This experiment resulted in the identification of 32 genes significantly higher expressed in pericycle cells compared to the non-pericycle cells (Dembinsky et al., 2007). These genes belonged to multiple functional classes including transcription, protein synthesis, disease/defense, signal transduction, metabolism, protein fate and subcellular localization. To identify genes preferentially expressed in pericycle cells that might not have been included in the 12 k microarray chip used in this study, suppression subtractive hybridization (SSH) and sequencing of expressed sequence tags (ESTs) were performed (Dembinsky et al., 2007). SSH led to the identification and validation of seven genes preferentially expressed in pericycle cells belonging to the functional categories of protein synthesis, cellular transport, disease/defense, signal transduction and metabolism. Moreover, 377 ESTs were sequenced from pericycle and 324 from non-pericycle central cylinder cells. Among those, the category protein synthesis was overrepresented and the category cell fate was underrepresented in pericycle compared to non-pericycle central cylinder cells (Dembinsky et al., 2007).

To identify distinct and shared components of the lateral root initiation pathway in maize primary and adventitious roots, pericycle cells were isolated via LCM from the different root types at multiple time points using a lateral root inducible system (Jansen et al., 2013). The authors discovered a shared core regulation system between primary and adventitious roots. This regulatory system was shown to be enriched for genes of the functional categories DNA binding and microtubule movement, indicating functions in gene regulation and cell division (Jansen et al., 2013). Moreover, in this study 14 genes from monocot-specific gene families, which might be involved in the positioning of lateral root initiation, were discovered. These genes might be involved in the different positioning of lateral root founder cells between Arabidopsis and maize (Jansen et al., 2013). This analysis also led to the identification of root type-specific transcription factors, of the MYB and MYB-related, homeobox and bHLH families (Jansen et al., 2013). A comparative study of maize (Jansen et al., 2013) and previously published Arabidopsis (Vanneste et al., 2005; De Smet et al., 2008) transcriptome datasets led to the identification of a shared core set of 60 genes. Moreover, these authors compared the phloem pole pericycle cell transcriptome of maize with transcriptomic profiles of xylem and phloem pole pericycle cells in Arabidopsis (Jansen et al., 2013). They showed that multiple genes including the gene *LBD16/ASL18* were underrepresented in phloem pole pericycle cells of Arabidopsis, while the corresponding orthologs were induced upon lateral root induction in phloem pole pericycle cells of maize (Jansen et al., 2013). These findings indicated a shared identity between xylem pole pericycle cells of Arabidopsis and phloem pole pericycle cells of maize (Jansen et al., 2013). In addition, the existence of a conserved regulatory network among maize and Arabidopsis was further substantiated by the observed intersection of expression profiles of auxin-related genes (Jansen et al., 2013).

## Cell Type-Specific Responses to Nitrate Linked to Lateral Root Formation

Lateral roots shape overall root architecture based on the availability of nutrients such as nitrate and phosphate (Araya et al., 2014; Péret et al., 2014). Genotypes with sparsely spaced and long lateral roots are optimal for nitrate acquisition, while genotypes with densely spaced and short lateral roots are optimal for phosphate acquisition in crops (Lynch, 2011, 2013). Cell type-specific experiments have been performed to uncover transcriptomic changes in different cell types, which might regulate the adaptation observed upon nitrate treatment. Cell type-specific transcriptome analyses showed that nitrogen responses are to a large extent cell type-specific (Gifford et al., 2008). One of these cell type-specific nitrogen response genes, *ARF8*, which has been linked to root development before (Gutierrez et al., 2009), was induced in pericycle cells through nitrogen (Gifford et al., 2008). Furthermore, *miR167a* was down regulated in pericycle cells in response to nitrogen treatment, resulting in the up regulation of *ARF8*, which is a target of *miR167a* (Gifford et al., 2008; Gutierrez et al., 2009).

Similarly, nitrate treatment induced the expression of the *Auxin Receptor F-Box Protein 3* (*AFB3*), a target of *miR393* in pericycle cells of Arabidopsis (Vidal et al., 2010). This suggests that both are part of a network regulating lateral root formation as a response to high nitrate availability. Later, it was demonstrated that *AFB3* regulates the expression of NAM/ATAF/CUC transcription factor NAC4 and DNA-binding-with-one-finger (*DOF*) transcription factor binding protein 4 (OBP4) upon nitrogen treatment (Vidal et al., 2013). This emphasizes the existence of a regulatory module, which acts downstream of an auxin signal leading to the initiation of lateral roots through regulation of cell cycle progression in the pericycle (Vidal et al., 2013). This is in line with previous reports of *OBP4* gene function in cell cycle regulation in Arabidopsis (Skirycz et al., 2008).

By utilizing FACS and GFP marker lines specific to Arabidopsis xylem pole pericycle cells, it was possible to observe the response of this cell type under nitrate influence over a time period of 48 h (Walker et al., 2017). In this experiment, NITRITE REDUCTASE (NIA1) and other NITRATE TRANSPORTERS (NRT2.3 and NRT2.5) were down regulated in the pericycle as an immediate response to the treatment (Walker et al., 2017). In the same context, the gene *SENESCENCE-ASSOCIATED GENE 21 (SAG21)*, which has been previously linked to regulation of lateral root development and biotic responses (Salleh et al., 2012), was shown to be down regulated in the pericycle cells as a late response to nitrogen (Walker et al., 2017). In contrast, *WRKY75*, previously reported to regulate lateral root development (Devaiah et al., 2007), was strongly and directly up regulated in pericycle cells upon nitrogen treatment (Walker et al., 2017). Through the utilization of FACS and GFP-tagged lines, an induction of *TGA1*, *TGA4*, *NRT2.1*, and *NRT2.2* in pericycle cells in response to nitrate was observed, indicating a possible involvement in the regulation of nitrogen-induced lateral root initiation in Arabidopsis (Alvarez et al., 2014).

In maize phloem pole pericycle cells of shoot-borne brace roots, local high nitrate induces the expression of *B-type cyclin-dependent kinases* (*CDKB*) and *cyclin B* (*CYCB*) genes, while simultaneously inhibiting the expression of Kip-related proteins (Yu et al., 2015). *CDKB* and *CYCB* genes are positive regulators of cell cycle progression (Schnittger et al., 2002; Boudolf et al., 2004), while Kip-related proteins (KRPs) are repressors of the cell cycle (De Veylder et al., 2001), linking the observed expression pattern to a reactivation of the cell cycle in the pericycle (Yu et al., 2015). Furthermore, local nitrate influence led to a strong up regulation of *ZmPIN9* in phloem pole cells compared to endodermis and pericycle cells, indicating that this protein serves in the transport of auxin to adjacent pericycle cells (Yu et al., 2015). While *PIN*-formed genes have for long been known as auxin efflux carriers (Gälweiler et al., 1998), similar cell type-specific findings in rice emphasize that these monocot-specific *PIN* formed genes might also regulate the formation of the complex root systems developed by monocots (Wang et al., 2009; Yu et al., 2015).

Cell type-specific transcriptomics also enables comparisons between similar cell types in different root types within a species. In maize, it was shown that some nitrate-dependent expression patterns are conserved between the different root types (Yu et al., 2016a). Conversely, in brace roots the number of genes exclusively expressed in pericycle cells exceeded the amount of exclusively expressed genes in seedling root types under homogeneous low or local high nitrate conditions (Yu et al., 2016a). This indicates a root type-specific regulation of these genes during early lateral root initiation as well as a different response to nitrate influence (Yu et al., 2016a). Multiple nitrate-responsive genes specific to brace roots were shown to be involved in the assembly of nucleosomes/chromatin and in the microtubule motor activity needed for the movement of these during mitosis (Yu et al., 2016a).

## Conclusion and Prospects

Tissue and cell type-specific transcriptomics have promoted the understanding of the molecular networks involved in lateral root formation. Moreover, these studies identified regulators of lateral root formation and regulators of pericycle founder cell identity. Integration of transcriptome datasets was useful for mechanistic comparisons between dicots and monocots during lateral root development. In addition, cell type-specific transcriptomics accelerated a deeper understanding of nitrate induced functional networks of lateral root formation in Arabidopsis and maize at the cellular level. In the future, it will be desirable to integrate *in situ* root visualization techniques such as micro x-ray computer tomography with cell type-specific transcriptome analyses, although it is still challenging to apply FACS and LCM techniques to roots grown in natural soil environments. Single-cell RNA-seq (Birnbaum, 2018), will further increase the spatial and temporal resolution of cells involved in lateral root formation. This technique will for instance enable the identification of differences within pericycle cells that acquire lateral root founder cell identity and will reveal the details of the fine-tuned processes underlying lateral root organization and development.

## Author Contributions

AK, FH, and PY contributed to the writing of this review.

## Conflict of Interest Statement

The authors declare that the research was conducted in the absence of any commercial or financial relationships that could be construed as a potential conflict of interest.
